# Effects of acute sleep loss on diurnal plasma dynamics of CNS health biomarkers in young men

**DOI:** 10.1212/WNL.0000000000008866

**Published:** 2020-03-17

**Authors:** Christian Benedict, Kaj Blennow, Henrik Zetterberg, Jonathan Cedernaes

**Affiliations:** From the Departments of Neuroscience (C.B., J.C.) and Medical Sciences (J.C.), Uppsala University; Clinical Neurochemistry Laboratory (K.B., H.Z.), Sahlgrenska University Hospital; Department of Psychiatry and Neurochemistry (K.B., H.Z.), Institute of Neuroscience and Physiology, the Sahlgrenska Academy at the University of Gothenburg, Mölndal, Sweden; Department of Neurodegenerative Disease (H.Z.), UCL Institute of Neurology; and UK Dementia Research Institute at UCL (H.Z.), London, UK.

## Abstract

**Objective:**

Disrupted sleep increases CSF levels of tau and β-amyloid (Aβ) and is associated with an increased risk of Alzheimer disease (AD). Our aim was to determine whether acute sleep loss alters diurnal profiles of plasma-based AD-associated biomarkers.

**Methods:**

In a 2-condition crossover study, 15 healthy young men participated in 2 standardized sedentary in-laboratory conditions in randomized order: normal sleep vs overnight sleep loss. Plasma levels of total tau (t-tau), Aβ40, Aβ42, neurofilament light chain (NfL), and glial fibrillary acidic protein (GFAP) were assessed using ultrasensitive single molecule array assays or ELISAs, in the fasted state in the evening prior to, and in the morning after, each intervention.

**Results:**

In response to sleep loss (+17.2%), compared with normal sleep (+1.8%), the evening to morning ratio was increased for t-tau (*p* = 0.035). No changes between the sleep conditions were seen for levels of Aβ40, Aβ42, NfL, or GFAP (all *p* > 0.10). The AD risk genotype rs4420638 did not significantly interact with sleep loss–related diurnal changes in plasma levels of Aβ40 or Aβ42 (*p* > 0.10). Plasma levels of Aβ42 (−17.1%) and GFAP (−12.1%) exhibited an evening to morning decrease across conditions (*p* < 0.05).

**Conclusions:**

Our exploratory study suggests that acute sleep loss results in increased blood levels of t-tau. These changes provide further evidence that sleep loss may have detrimental effects on brain health even in younger individuals. Larger cohorts are warranted to delineate sleep vs circadian mechanisms, implications for long-term recurrent conditions (e.g., in shift workers), as well as interactions with other lifestyle and genetic factors.


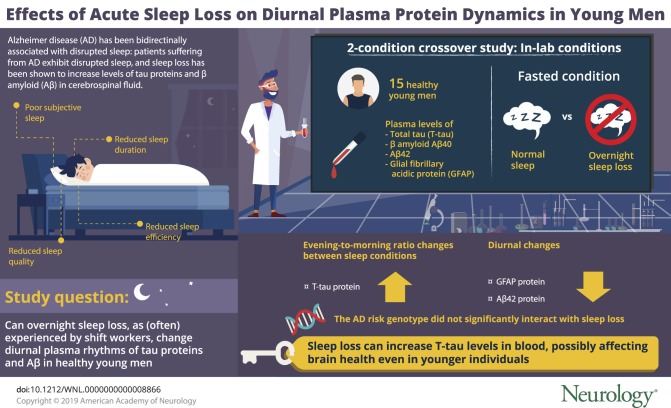


Whereas the importance of sleep for survival and cognitive performance has been recognized and studied for over a century,^1,2^ only during the last decade has research begun to mechanistically unveil the bidirectional relationship between sleep and long-term health of the CNS.^3^ The most common form of dementia, Alzheimer disease (AD), is hallmarked by the pathologic accumulation of both extracellular β-amyloid (Aβ) and intracellular tau proteins in amyloid plaques and neurofibrillary tangles, respectively. AD has been associated with poor subjective sleep, as well as reduced sleep duration, sleep quality, and efficiency, in what appears to constitute a bidirectional relationship.^3–6^ As such, this can be speculated to be able to develop into a cycle.^3^ Even so, there is also evidence to suggest that disrupted sleep as in obstructive sleep apnea does not increase the risk of cognitive decline,^7^ prompting the need for more experimental and longitudinal studies.

In humans, acute sleep loss increases levels of Aβ species and total tau (t-tau) in CSF.^3,8^ Furthermore, patients with obstructive sleep apnea exhibit increased Aβ levels in plasma, as well as higher levels of phosphorylated tau.^9^ The underlying mechanism may be a reduced functionality of the glymphatic system, which in mice has been shown to primarily function during sleep, to increase clearance of interstitial metabolites such as Aβ peptides from the brain parenchyma.^3^ Furthermore, sustained wakefulness as occurs during jet lag, shift work, and chronic sleep loss results in increased duration of the time when neurons are firing at a higher frequency, and neuronal activity is known to increase local CNS production or release of Aβ and tau,^3,8^ demonstrating an intricate link between neuronal metabolic state and both the production and clearance of the key molecular substrates in the pathogenesis of AD.

Several blood-based AD biomarkers exhibit strong correlations with CSF levels,^10^ and may thus be of practical use for clinical purposes. As of yet, it is unknown whether observed increases in biomarkers such as tau in CSF also are reflected by altered blood levels. It is also unclear whether such biomarkers exhibit the same diurnal pattern in blood in response to sleep loss, and whether these dynamics depend on genetic factors.^8^

Against this background, our aim was to assess the effect of acute sleep loss on diurnal plasma rhythms of t-tau and Aβ species in healthy young humans. Furthermore, as previous studies have provided evidence for disruption of both glial and neuronal cells in response to experimental sleep loss,^11^ we also aimed to investigate biomarkers of neuroaxonal injury. We thus also assessed levels of neurofilament light chain (NfL), a protein found in axons that is released following white matter injury and degeneration, as well as glial fibrillary acid protein (GFAP), which also is elevated in AD.^10^

## Methods

Eligible participants were invited during 2012–2013 to participate in a within-subject crossover design study. Seventeen participants were deemed eligible and chose to participate in the study. Out of these, one participant was unable to complete his second session due to scheduling conflicts. Another participant was unable to sleep during his sleep session and was therefore excluded. Thus, 15 healthy normal-weight men (age 22.3 ± 0.5 years, body mass index 22.6 ± 0.5 kg/m^2^, as reported in reference 12) participated in both nighttime interventions, in randomized order, i.e., a night of normal sleep compared with a night of sleep loss. Study size was chosen based on similar studies in the field of biomarker analysis related to neuronal health (c.f. references 11 and 13) and the field of sleep metabolism (c.f. reference 14).

Prior to enrollment, participants answered questions regarding their general, metabolic, neurologic, and psychiatric health, as well as the potential use of medications and drugs. This was subsequently followed up by interview (J.C.). Only self-reportedly healthy participants, who were free from chronic medication or current or prior drug use, were included. Participants furthermore had to have a self-reported sleep duration of 7–9 hours per night, have good sleep quality (Pittsburgh sleep quality index ≤5), and be regular breakfast eaters. Exclusion criteria were extreme chronotypes (assessed by the Morningness-Eveningness Questionnaire), any form of smoking, nicotine use, or alcohol intake greater than 5 standard units per week. Additional exclusion criteria included shift work within the last 6 months or traveling across 1 or more time zones within 3 weeks of scheduled participation in the study or between study sessions.

The week prior to participating in each session, participants maintained sleep/wake diaries, which confirmed that participants had the same amount of sleep the week prior to each condition (as reported in reference 12). Furthermore, participants slept a single night in the laboratory within 2 weeks of participating in their first session, in order to ensure habituation to the laboratory conditions. Participants arrived at the laboratory at Uppsala University Biomedical Center 2 evenings prior to the overnight intervention. While in the laboratory, participants stayed in their own individual rooms with room lights on (∼250 lux in the direction of gaze when standing or sitting), were continuously monitored, and were instructed to remain sedentary apart from 2 supervised 15-minute-long walks during the morning and afternoon. Throughout each in-laboratory day, 3 isocaloric meals (at 08:00, 13:00, and 20:00 hours) were provided, and each meal had to be consumed within 20 minutes. After a baseline day and night in our laboratory, blood was taken in the fasted condition at 19:30 hours. Following the scheduled dinner at 20:00 hours, the nighttime intervention took place between 22:30 and 07:00 hours. Participants were unaware of the experimental overnight condition until being informed at 21:00 hours of each intervention night. In the sleep condition, participants were allowed to sleep 22:30–07:00 hours in 0 lux. During this, sleep was recorded by EEG, EMG, and electrooculography, which was subsequently scored according to standardized criteria by a scorer blinded to the study order and study hypothesis (sleep session sleep duration: 483 ± 3 minutes [as reported in reference 12], median 485.5, range 460–490 minutes; sleep efficiency: median 96.1, range 93%–99%). In the sleep loss condition, lights were kept on during the 22:30–07:00 hours intervention period, and participants were bed-restricted to approximate activity levels during the corresponding sleep condition. During the continued wakefulness, participants sat in semirecumbent position in their bed with their backs supported, and were allowed to play board games, watch movies, and engage in discussions. During this time, participants were under continued constant direct visual supervision by the experimental leaders to ensure sustained wakefulness. Furthermore, water but not food intake was allowed, and light levels were kept on at 250 lux in the direction of gaze. The morning following each intervention, blood was once more drawn in the fasted state at 07:30 hours.

### Standard protocol approvals, registrations, and patient consents

The study was approved by the Uppsala regional ethical board (approval 2012/477/1), and all participants provided oral and written informed consent. The study is registered at Clinicaltrials.gov (NCT01800253).

### Plasma separation and biochemical analyses

Blood was drawn into PSTII tubes (BD Sweden, Stockholm), followed by centrifugation (1,300 rcf, 10 minutes, 4°C), and aliquoting of the plasma supernatant that was frozen on dry ice, for subsequent storage at −80°C until analysis. Plasma levels of t-tau, Aβ40, and Aβ42 were analyzed using commercially available ultrasensitive single molecule array (Simoa) assays (Quanterix, Lexington, MA). Plasma NfL levels were determined using an in-house ultrasensitive Simoa assay, as previously described in detail.^15^ Each participant’s samples were measured in succession to avoid batch effects. All analyses were performed by board-certified technicians, who were experts in biomarker assays and who were blinded to clinical data, using detailed standardized procedures to assure analytical precision.^16^

### Genotyping

The Illumina (San Diego, CA) OmniExpressExome array was used to genotype the participants, followed by analysis with the software PLINK version 1.9 to determine polymorphisms at the genotyping at single nucleotide polymorphism (SNP) rs4420638.^17^ The OmniExpressExome array used for genotyping did not contain SNP data for the canonical SNPs (rs7412 and rs429358) used to determine the *APOE*-specific genotype that has been linked to increased risk of AD, but rs4420638 also has been found to reflect an increased risk of AD (in one study noted as a ∼4 times greater risk^17^).

### Statistics

Prism (v 8.2) was used for all statistical analyses with the exception of Bayes factor (BF), which was calculated in R (v 3.5.1), using the package BayesFactor (v 0.9.12). This computes the BF via Gaussian quadrature, and the default *r* scale factor √2/2 was used. We calculated the BF as an alternative statistical approach: a BF of 1 is seen as no evidence for the H_1_ hypothesis; a value of 1–3 or 1/3–1 is seen as anecdotal evidence, 3–10 or 1/3–1/10 as moderate evidence, and 10–30 or 1/10–1/30 as strong evidence. Normal distribution was determined using the Shapiro-Wilk test (*p* > 0.05). As we had large interindividual differences in absolute levels of the analyzed factors, we opted to use ratios between evening and morning values, and then compared these ratios between our 2 sleep conditions. For normally distributed data, the paired Student *t* test and Pearson correlations were used. The morning vs evening difference across conditions of Aβ40, Aβ42, and GFAP levels were analyzed using the one-sample Student *t* test (parametric data) or the Wilcoxon signed rank test (nonparametric data) for the full set of samples (pooled for both experimental conditions). The genotype effect for evening vs morning changes in Aβ40 and Aβ42 levels between the sleep conditions in carriers vs noncarriers of rs4420638 was assessed by determining the genotype × sleep condition interaction in a 2-way analysis of variance. We were able to determine plasma levels across the 2 timepoints and 2 conditions for GFAP in 15/15 participants, t-tau in 13/15 participants (2 participants had single missing values), NfL in 13/15 participants (2 participants had consistent plasma NfL levels >100 times greater than the other participants' averages in NfL, indicating presence of heterophilic antibodies), Aβ40 in 9/15 participants (1 participant was excluded due to more than a single missing value), and Aβ42 in 11/15 participants (1 participant was excluded due to more than a single missing value). When only one value was missing for a given individual and analyte, data were imputed, corresponding to 2 single timepoints for t-tau, 5 for Aβ40, 3 for Aβ42, 0 for NfL, and 0 for GFAP.

### Data sharing statement

Anonymized original data will be shared by request from any qualified investigator after inquiry. This includes raw analytical parameters, files used for statistics, and R code, but not the study protocol.

## Results

**Figure 1:**
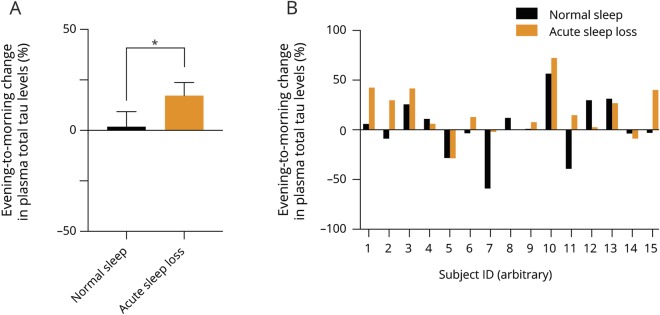
Effect of acute sleep loss on diurnal changes in plasma levels of total tau (t-tau) (A) Change in plasma levels of t-tau, comparing the percent change from evening at 19:30 hours to the morning at 07:30 hours, between the normal sleep (absolute levels in the evening: 3.87 ± 0.52 pg/mL) and acute sleep loss condition (evening absolute levels: 3.00 ± 0.42 pg/mL) in 15 healthy young men. (B) The evening to morning change in plasma levels of t-tau across individuals, in response to normal sleep (black bars) and acute sleep loss (orange bars) (individuals arbitrarily numbered along the x axis; n = 15). *p < 0.05; grouped values shown as mean ± SEM.

**Figure 2:**
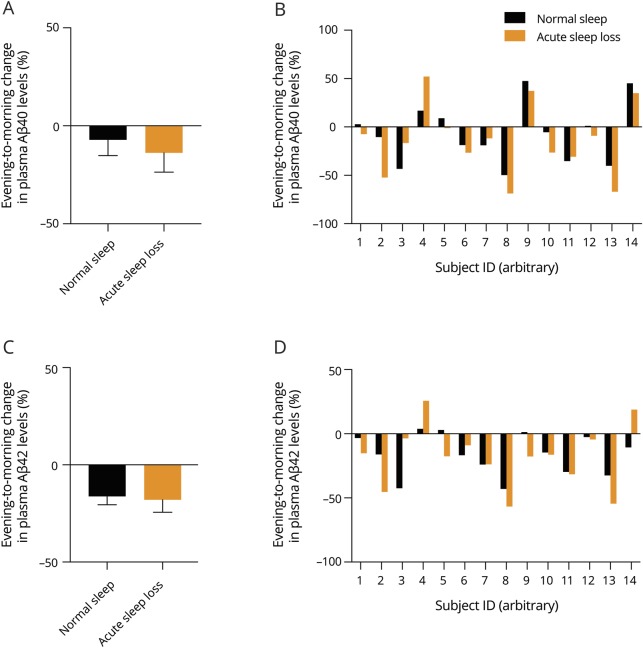
Effect of acute sleep loss on diurnal changes in plasma levels of amyloid β40 (Aβ40) and amyloid β42 (Aβ42) (A) Change in plasma levels of Aβ40 from evening to 12 hours later in the morning (expressed as % change), after each of 2 overnight interventions, i.e., normal sleep (absolute levels in the evening: 29.9 ± 2.3 pg/mL) and acute sleep loss (evening: 28.3 ± 2.3 pg/mL) (n = 14). (B) Data from A shown at the individual level (participants arbitrarily numbered along the x axis), as the evening to morning change, in response to normal sleep (black bars) and acute sleep loss (orange bars). (C) Corresponding evening to morning change at the group level for plasma levels of Aβ42 (n = 14), across normal sleep (evening: 2.63 ± 0.13 pg/mL) and sleep loss (evening: 2.98 ± 0.16 pg/mL); also shown in D at the individual level (as in B).

We first assessed evening to morning changes across sleep conditions for levels of t-tau protein. Similar to recent findings based on CSF sampling under conditions of acute sleep loss,^8^ the evening to morning change in average plasma levels of tau exhibited an increase following sleep loss, compared with normal sleep (*p* = 0.0345; BF = 2.05) (figures 1 and 2, A and B).

We next assessed whether sleep loss would alter overnight changes in plasma levels of Aβ species, but observed no significant evening to morning changes in Aβ40 (*p* = 0.24; BF = 0.51) or Aβ42 (*p* = 0.76; BF = 0.28) following acute sleep loss compared with sleep (figure 2, A–D). Given that certain SNPs in apolipoprotein genes have been linked to an increased risk of AD, we also set out to interrogate whether those at increased genetic risk of AD also have altered Aβ dynamics in response to sleep loss, a largely unexplored area of inquiry.^18^ In this context, the G and G/G variant of the SNP rs4420638 (within the apolipoprotein gene *APOC1*) have been associated with an increased risk of AD,^17,19^ by being in linkage disequilibrium with the canonical AD risk gene *APOE*,^17^ and thus affecting metabolism of Aβ species. However, we found no significant interaction effects between the genotypes (7 of 14 participants were G or G/G carriers) of rs4420638 and the evening to morning change in plasma levels of Aβ40 (*p* = 0.63) or Aβ42 (*p* = 0.37) in response to sleep loss compared with normal sleep.

Previous studies have provided evidence for changes in sleep measures and AD biomarkers (such as for tau, c.f. reference 5). We therefore also assessed whether sleep efficiency in the sleep condition correlated with the overnight change in plasma levels of t-tau, Aβ40, or Aβ42, but found no evidence in support of this notion in our smaller set of individuals (all *r* < 0.42; *p* > 0.10).

**Figure 3:**
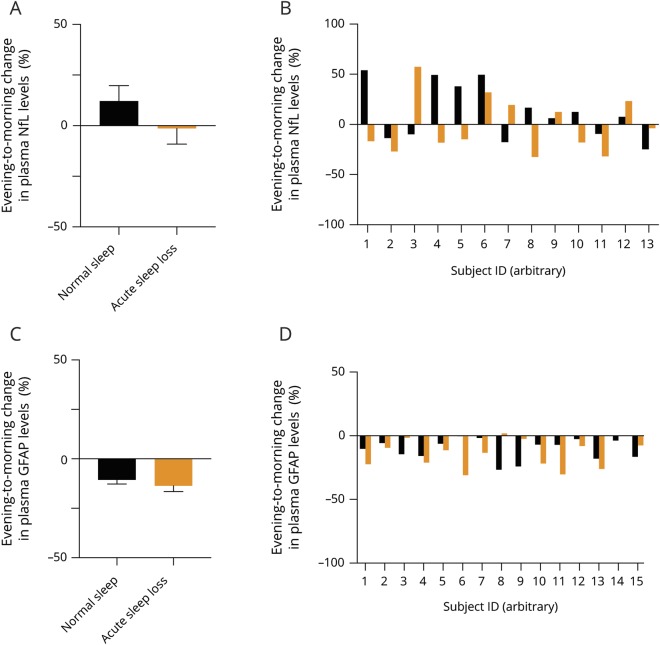
Effect of acute sleep loss on diurnal changes in plasma levels of neurofilament light chain (NfL) and glial fibrillary acid protein (GFAP) (A) Evening to morning change (expressed as % change) in plasma levels of NfL after normal sleep (absolute levels in the evening: 14.9 ± 2.0 pg/mL), and acute sleep loss (evening: 15.2 ± 2.3 pg/mL) (n = 13). (B) Individual evening to morning changes across the studied participants in plasma levels of NfL in response to normal sleep (black bars) or acute sleep loss (orange bars). (C) Change in plasma levels of GFAP in evening to morning after normal sleep (evening: 120 ± 50 pg/mL) and acute sleep loss (evening: 115 ± 42 pg/mL). (D) Individual evening to morning changes in plasma levels of GFAP in response to normal sleep and acute sleep loss (n = 15). Grouped values shown as mean ± SEM.

We furthermore did not observe any significant differences between the 2 experimental conditions when we compared how they affected the evening to morning change in plasma levels of the neuroaxonal damage markers NfL (*p* = 0.26; BF = 0.49) (figure 3, A and B) or GFAP (*p* = 0.48; BF = 0.33) figure 3, C and D).

**Figure 4:**
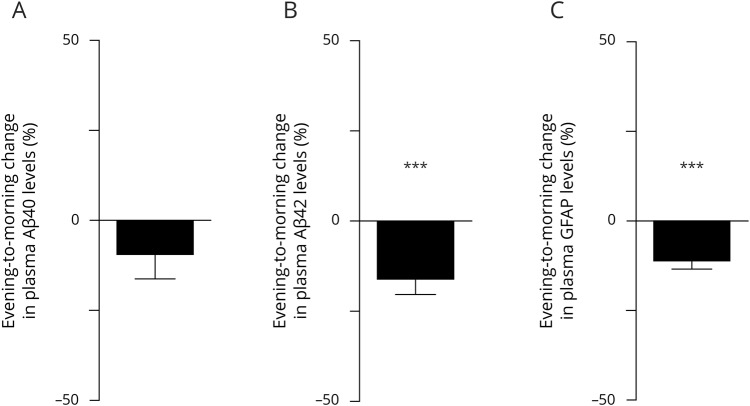
Analysis of diurnal changes across sleep conditions in plasma levels of amyloid β40 (Aβ40), amyloid β42 (Aβ42), and glial fibrillary acid protein (GFAP) Evening to morning change (expressed as % change) in plasma levels of (A) Aβ40 (n = 28), (B) Aβ42 (n = 28), and (C) GFAP (n = 30). Samples were pooled across conditions and are shown as mean ± SEM. ***p < 0.001.

Given that we observed uniform evening to morning directionalities at the group level across the experimental conditions for Aβ40, Aβ42, and GFAP in plasma, we proceeded to assess diurnal changes in plasma levels of these biomarkers. Whereas we noted no diurnal changes in plasma levels of Aβ40 (*p* = 0.107; BF = 0.68) (figure 4A), we noted significant evening to morning decreases in Aβ42 (−17.1%; *p* < 0.0001; BF = 223) and GFAP in plasma (−12.1%; *p* < 0.0001; BF = 104,161) (figure 4B-C). Altogether, these findings provide evidence that certain AD biomarkers exhibit diurnal changes that are sleep-independent.

## Discussion

Our present findings suggest that acute sleep loss—as often experienced in shift work, for example—results in an evening to morning increase in plasma levels of t-tau, without concomitant changes in plasma levels of other biomarkers of AD, such as Aβ species. Whether the changes in t-tau in plasma in response to sleep loss reflect increased neuronal activity during sustained wakefulness, or alternatively, disrupted central or peripheral clearance, is unclear.

A recent study described increased levels of tau in CSF of older adults undergoing overnight wakefulness.^8^ Our results suggest that this change in t-tau levels also is detectable at the peripheral level following acute sleep loss. A previous study by us, that is similar to the current one, found that acute sleep loss resulted in increased blood levels of markers of neuronal and astroglial injury.^11^ It is therefore possible that increased t-tau levels in blood could indicate sustained high neuronal activity during prolonged wakefulness,^20^ but it also may indicate increased neuronal damage under such conditions.^21,22^ This may result in increased leakage of tau into the interstitial fluid and subsequently into blood.^22^

Establishing how peripheral biomarkers of AD are controlled by diurnal vs sleep-related mechanisms can provide insight into the bidirectional sleep–AD relationship. For instance, a recent study in older participants has demonstrated that each SD increase in plasma tau is associated with a 35% greater risk of AD during subsequent follow-up.^23^ An important next step will be to firmly establish whether changes in tau related to sleep and its characteristics, as well as time of day, can be taken into account to assess future risk of AD. This could include the specific goal of establishing whether t-tau in plasma can be utilized to assess how participants with chronically poor sleep may be at a higher risk for clinically relevant neurodegeneration. Indeed, pathologic accumulation of Aβ and tau begins decades prior to clinical onset of AD, making early diagnosis and intervention of critical importance.

Previous studies have demonstrated that levels of Aβ exhibit a diurnal pattern in both blood and CSF, and that there exists a lag between the acrophase of each of these rhythms, such that the rhythm in plasma is delayed by about 6 hours.^24^ Clearance of Aβ across brain parenchyma has also been demonstrated to be highly dependent on the glymphatic system, such that Aβ clearance is twofold faster during sleep compared with wakefulness in mice.^25^ Furthermore, a study of 8 men of age 30–60 years found that scheduled sleep loss resulted in a ∼30% increase in CSF levels of Aβ38, Aβ40, and Aβ42.^26^ Overnight wakefulness has furthermore been reported to result in increased levels of Aβ in thalamus and hippocampus of 20 participants age 22–72 years, as assessed by PET, using the AD-specific tracer ^18^F-florbetaben.^18^ However, another study found no changes in the plasma Aβ42/Aβ40 ratio following 1 night of sleep loss in healthy humans who were not bed-restricted during sleep loss.^11^ With these previous studies in mind, our lack of findings at the peripheral level for overnight changes in Aβ peptides in response to sleep loss suggests that peripheral measurements of Aβ biomarkers may be less accurate in reflecting acute changes that occur in the CNS in response to sleep loss.

Another biomarker of relevance is NfL, levels of which increase in CSF of patients with AD and track well with cortical thinning and thus also with clinical progression of AD.^10^ These changes also parallel changes in blood.^10^ Given that we observed no sleep loss–induced changes in NfL (the most well-established neuronal injury marker) or GFAP, this argues against the possibility that acute sleep loss would also result in acute neuroaxonal injury. However, given that chronic sleep loss has also been independently associated with cognitive decline,^27^ it will be of interest to establish whether chronic or recurrent bouts of acute sleep loss affect plasma or CSF levels of NfL, to gain further insight into the mechanisms by which recurring sleep loss over time not only can promote neurodegeneration but also impair cognitive health.

The highly standardized study protocol utilized herein ensured that activity, meal, and light exposure were kept identical across the studied conditions, but the present study only included a small sample size of homogeneous, young men, all of whom were of normal weight and of good health. Given the small sample size, we cannot exclude the possibility that we were underpowered to detect smaller differences, such as diurnal or sleep loss–induced differences in levels of NfL, or differences related to the AD risk genotype that was investigated herein. This may especially have been the case as we—similar to that previously reported by Lucey et al.^26^—observed overall large interindividual differences for the investigated analytes. Furthermore, even though changes in plasma track changes in CSF for markers such as NfL,^10^ the absence of CSF sampling in our study precludes any assessment regarding how these markers may have been altered in CSF under the present study conditions. It is also important to note that we cannot rule out that peripheral mechanisms such as increased breakdown may have contributed to the lack of sleep loss–related changes in peripheral plasma levels, such as for Aβ42. Studies aimed at examining the dynamic relationship between CSF and plasma levels of Aβ species under various conditions of sleep loss will hopefully be able to establish to what extent peripheral clearance of Aβ species is affected by disrupted sleep/wake cycles. Of equal importance will be to stablish how the acute changes we and others have observed in response to acute sleep loss relate to recurrent bouts of prolonged wakefulness (e.g., in shift workers), and similarly, to the more long-term associations that have been established by us and others between sleep disruption and future risk of AD.^4^ Finally, given that we only collected blood samples in the early evening and morning, it is possible that additional blood sampling may reveal additional dynamics of how CNS health biomarkers are affected by sleep loss and circadian misalignment.

Our observation of altered evening to morning levels of t-tau in blood in response to sleep loss is intriguing in light of our previous findings that demonstrated increased blood levels of neuron-specific enolase and S100 calcium-binding protein B after 1 night of sleep loss, also in healthy young individuals.^11^ Thus, our findings indicate that even at a young age, sleep loss may alter CNS processes in a direction that promotes neuronal damage and AD pathogenesis. It should be noted that another study found no changes in AD biomarkers in CSF, including Aβ species, t-tau, and phospho-tau, following recurrent partial sleep restriction (to 4 hours/night) in healthy young individuals.^28^ This suggests that changes may depend on a variety of factors, such as participant characteristics (including age), as well as the type, acuteness, and cumulative duration of sleep loss. Indeed, another study found that specific disruption of slow-wave sleep (which is largely conserved under conditions of partial sleep restriction) resulted in increased levels of Aβ40 in CSF.^29^ Slow-wave sleep declines with advanced age, and separately, reduced non-REM sleep (which encompasses slow-wave sleep) has been associated with increased CNS tau pathology.^5^ Another potential mechanism may be that conferred by genetic factors, and at present it is poorly understood whether genetic risk factors that alter metabolism of Aβ species may increase AD pathogenesis by interacting with sleep and circadian mechanisms.

Our study only examined healthy young men without any long-term or recent history of shift work. Our findings may therefore not translate to women, older individuals, or people who are already at risk of adverse effects associated with disrupted and misaligned sleep. This includes individuals who already are carrying out shift work, as well as patients with obesity or diabetes.^30,31^

Further studies are warranted to assess whether the changes we observed are modulated by factors such as measures of sleep quality, age, or metabolic disease,^31^ and whether there also are protective genetic factors that may further modulate how sleep loss may promote neurodegenerative processes. Indeed, given that a variety of lifestyle factors other than sleep are able to modulate the risk of dementia and AD,^32^ it will now be important to disentangle how such lifestyle factors interact with sleep/wake patterns to modify the long-term risk of AD, with the goal of improving public health and reducing the burden of AD to society. For instance, it can be argued that prophylactic measures to lower the risk of AD—especially in groups at higher risk such as those with genetic predisposition to develop AD—should also include sleep hygiene.

Taken together with previous studies that also have examined changes in AD biomarkers in CSF,^8,13^ our findings provide mechanistic insight into how disruption of sleep or circadian rhythms may lead to an increased risk of cognitive decline and AD.^3,27^ Our exploratory findings uncover that acute sleep loss results in altered diurnal plasma dynamics of a key pathogenic biomarker of AD. We also find preliminary evidence for diurnal changes in biomarkers utilized in neurodegenerative analyses, suggesting that care should be taken when considering the time of day that such analytes are sampled. Our results are based on a fairly small sample size and only focused on changes following an acute, yet highly controlled, sleep intervention. However, taken together with previous findings, our results provide further preliminary evidence to suggest that even in young, healthy individuals, sleep is not only of relevance for cognitive performance, but also may matter for long-term brain health. Given the long latent onset of AD and the accumulating evidence for how disrupted sleep/wake rhythms can impair brain health in young individuals, further longitudinal studies and randomized controlled trials are warranted to establish whether curtailed, disrupted, or misaligned sleep increase the risk of dementia. Altogether, this could provide key insight into whether interventions targeting sleep should be initiated at an early age to reduce the risk of dementia and AD.

## Glossary

Aβ: β-amyloid
AD: Alzheimer disease
BF: Bayes factor
GFAP: glial fibrillary acid protein
NfL: neurofilament light chain
SNP: single nucleotide polymorphism
t-tau: total tau
